# Effect of Hypoxia-Induced MicroRNA-210 Expression on Cardiovascular Disease and the Underlying Mechanism

**DOI:** 10.1155/2019/4727283

**Published:** 2019-05-21

**Authors:** Yinuo Guan, Xianjing Song, Wei Sun, Yiran Wang, Bin Liu

**Affiliations:** Department of Cardiology, The Second Hospital of Jilin University, Changchun, Jilin, China

## Abstract

Cardiovascular diseases have high morbidity and mortality rates worldwide, and their treatment and prevention are challenging. MicroRNAs are a series of noncoding RNAs with highly conserved sequences and regulate gene expression by inhibiting mRNA transcription or degrading targeting proteins. MicroRNA-210 is significantly upregulated during hypoxia and plays a protective role by inhibiting apoptosis and regulating cell proliferation, differentiation, migration, mitochondrial metabolism, and angiogenesis in hypoxic cells. MicroRNA-210 expression is altered in cardiovascular diseases such as atherosclerosis, acute myocardial infarction, preeclampsia, aortic stenosis, and heart failure, and overexpression of microRNA-210 in some of these diseases exerts protective effects on target organs. Furthermore, chronically upregulated miR-210 potentially plays a marked pathogenic role in specific situations. This review primarily focuses on the upstream pathways, downstream targets, clinical progress in cardiovascular disease, and potential applications of microRNA-210.

## 1. Introduction

Cardiovascular diseases (CVDs) currently have the highest morbidity and mortality rates worldwide, in both developing and developed countries, and the number of lives lost increases every year [1]. According to recent statistics from the American Heart Association on heart disease and stroke, 17.3 million people die every year from CVD-related complications globally [2]. In addition to continuous research on the prevention and treatment of CVD, the development of strategies for improving the quality of life and prognosis of patients with CVD has recently emerged as an important avenue of study.

MicroRNAs (miRNAs) are small molecules of 20–26 nucleotides with a highly conserved sequence of single chain-encoded RNA [3]. Accumulating evidence indicates that miRNAs regulate many biological processes such as cell proliferation, differentiation, apoptosis, autophagy, mitochondrial metabolism, angiogenesis, tumor formation, and hematopoiesis [4, 5]. These regulatory effects are largely achieved by destabilizing target mRNAs or inhibiting translation [6]. To date, more than 700 species of miRNAs that regulate 20–30% of all protein-coding genes in the human body have been identified [3]. Among these, microRNA-210 (miR-210) is a well-known hypoxia miRNA, which leads to similar changes in most cell lines [7]. miR-210 is upregulated in normal cells exposed to hypoxia and in hypoxic tumor cells and has shown great therapeutic potential in various diseases [8–11]. Studies have established the role of miR-210 in almost all hypoxia-related phenomena, such as angiogenesis, apoptosis, differentiation, proliferation, cell cycle regulation, mitochondrial metabolism, DNA damage repair, and tumor growth [12].

Although numerous genes are regulated by miR-210, only a few have a protective function against CVD [13–16]. Most of the products of these genes mediate the protective response of the cardiovascular system to hypoxia and improve the adaptability of cells and individuals to hypoxia through various biological functions. Furthermore, miR-210 potentially enhances the progression of some chronic diseases, such as pulmonary arterial hypertension (PAH) [17]. Here, we review the most recent studies on miR-210 and its expression patterns in relation to CVD. Based on the identified target genes of miR-210 and associated pathways and functions, we highlight the prospects of miR-210 as a target for future treatment and prevention of CVD.

## 2. miR-210 and Its Activators

The most common and prominent cause of CVD is hypoxia, which leads to irreversible tissue damage [18]. Kulshreshtha et al. and Camps et al. first reported that miR-210, located in the intronic sequence *AK123483* mRNA, is hypoxia-inducible [11, 19]. Many subsequent studies have since confirmed that miR-210 expression can be induced by hypoxia, which is triggered by the expression of hypoxia-inducible factor-alpha (HIF-*α*) including HIF-1*α* and HIF-2*α* [8, 20–22]. The HIF molecule is prolyl-hydroxylated by three homologous 2-oxoglutarate-dependent dioxygenases PHD1, PHD2, and PHD3 and ubiquitinated and rapidly degraded by the von Hippel-Lindau (VHL) protein under normal conditions [23, 24]. HIF-1*α* accumulates in most cell lines owing to inhibition of PHD during hypoxia conditions, followed by translocation to the nucleus, wherein it interacts with aromatic hydrocarbon nuclear transfer protein (ARNT) [25, 26]. HIF-1*α* binds to the miR-210 promoter at approximately 40 bp upstream of the transcription initiation site and upregulates miR-210. Consistently, Kulshreshtha et al. confirmed that anti-HIF-1*α* antibody immunoprecipitated the miR-210 promoter fragments in hypoxic cells, albeit to a minor extent in normoxic controls [11]. Furthermore, miR-210 was reportedly downregulated in *HIF-1α* knockout mice, compared with the wild-type mice [27]. Wang et al. [28] analyzed infarcted heart tissues from patients who died of acute myocardial infarction (AMI), reporting that miR-210 was significantly upregulated in the infarcted samples compared with those of the control group (accidental death). Interestingly, HIF-1*α* was downregulated in the AMI samples, suggesting that miR-210 may function as an HIF1-*α* inhibitor.

Although previous studies reported that the regulation of hypoxia-response element (HRE) with miR-210 displays specificity towards HIF-1*α* rather than HIF-2*α* [20], some studies reported that miR-210 may be upregulated via an HIF-1*α*-independent pathway recently. Zhang et al. [29] used RNA interference to silence HIF-2*α* in 786-O-pBABE cells, which are predominantly HIF-2*α*-dependent for HIF activity, and reported significant silencing of miR-210. Similarly, Kulshreshtha et al. [11] investigated the effect of exogenous constitutively active HIF-2*α* in several tumor cells and reported that miR-210 is upregulated. The HIF-2*α* pathway depends on dimerization with ARNT, and a deletion of ARNT results in complete inactivation of the HIF pathway.

However, miR-210 was also upregulated in the absence of a complete HIF signal conduction in fibroblasts of aromatic ARNT-knockout mouse embryos, indicating that miR-210 is regulated by other pathways in addition to HIF-1*α* or HIF-2*α* [10]. p53, which also directly binds to the miR-210 promoter, is a well-known posttranslational regulator of protein degradation in response to stress, including hypoxia. It regulates important cellular activities, such as the cell cycle and apoptosis, and also suppresses inflammation in various human tissues [30]. p53 reportedly plays a role in miRNA processing through interaction with Dicer and is an important component for pre-miRNA processing to fully active miRNA [31]. Regulation by p53 suggests that miR-210 also plays a protective role in inflammation or other stimulations besides hypoxia. In addition to the p53 pathway, the AKT pathway plays an important role in regulating miR-210. Kim et al. [32] reported that the AKT and ERK1/2 pathways promote miR-210 through an HIF-1*α*-independent pathway. Shi et al. [33] reported that insulin can stimulate miR-210 expression via the PI3K/AKT pathway. Stimulation of AKT expression potentially upregulates miR-210 [10]. Furthermore, NF-*κ*B also induces miR-210 expression under hypoxia. Subunit p50 of NF-*κ*B, a protein related to inflammation and DNA repairing, localizes at a 200 bp core promoter region immediately upstream of the miR-210 stem-loop. Sequence analysis of NF-*κ*B p50 binding with miR-210 was also reported by Zhang et al. [34]. Moreover, serum response factor (SRF), as an exogenous miRNA promotor, can regulate several miRNAs, including miR-210. Chromatin immunoprecipitation (ChIP) revealed a direct interaction between SRF and miR-210, which inhibited the proliferation and differentiation of cardiac progenitor cells in embryonic stem cell-derived embryoids [35].

## 3. Clinical Relevance of miR-210 in CVD

Similar to HIF-1*α*, p53, AKT, and NF-*κ*B serve as protective components in various diseases in the cardiovascular system [10, 28, 33, 36]. Studies using animal models of CVD have indicated a protective role of miR-210. In AMI rats, miR-210 upregulation and adenomatous polyposis coli (APC) downregulation can be observed in cardiomyocytes (CM), suggesting that APC potentially participates in the reduction of cell death and promotion of angiogenesis via miR-210 regulation [21]. Li et al. [15] established a mouse model of atherosclerosis through administration of a high-fat diet, wherein miR-210 was significantly upregulated and the proapoptotic protein, 3-phosphoinositide-dependent protein kinase-1 (PDK1), was inhibited. Moreover, the significant upregulation of vascular endothelial growth factor (*VEGF*) and hepatocyte growth factor (HGF) in miR-210 overexpressing mice clearly suggests an increased angiogenic potential of miR-210 [37–39]. In CVD, miR-210 is believed to protect the cardiovascular system from potentially lethal damage by inhibiting cell apoptosis and promoting angiogenesis, thus potentially leading to revascularization.

**Figure 1:**
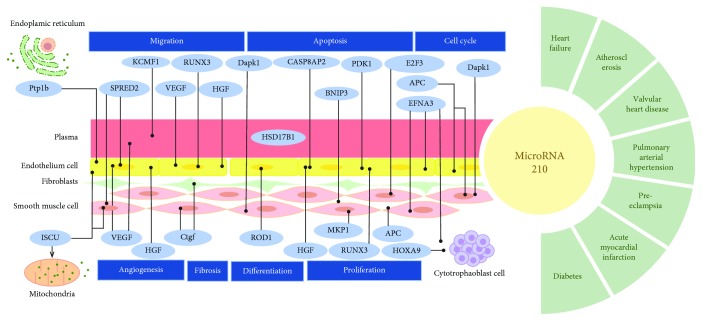
Genes and CVD related to miR-210.

Although no clinical study has reported the precise association between miR-210 and cardiovascular protection, alterations in miR-210 expression are often observed in peripheral blood or damaged tissues from patients with CVD, including atherosclerosis, acute coronary syndrome, valvular heart disease, pulmonary arterial hypertension, and heart failure. Moreover, changes in miR-210 expression have been reported in some cardiovascular-associated diseases, such as preeclampsia and diabetes. These changes are associated with typical genes or proteins involved in cardiovascular damage or repair (Table 1). Hale et al. [40] confirmed Argonaute 2 as a major regulator of specific circulating miRNAs under dynamic conditions, released together with circulating miR-210 under hypoxic conditions. Argonaute 2 potentially facilitates miR-210 delivery to recipient cells and protects individuals exposed to hypoxia. Thus, a consensus regarding the potential applications of miR-210 to evaluate disease severity and predict disease prognosis has been established (Figure 1).

### 3.1. Atherosclerosis

Atherosclerosis is characterized by chronic inflammation, which not only is limited to the heart but also involves peripheral vessels, causing stenosis in the femoral arteries and carotid arteries. Raitoharju et al. [53] analyzed samples obtained from carotid and femoral endarterectomy or in coronary artery bypass grafting with a PCR array and reported that the level of miR-210 was increased significantly and that one of its downstream target genes, potassium channel modulatory factor 1 (*KCMF1*), was significantly downregulated. Another study reported that miR-210 levels were significantly increased in blood samples from patients with arteriosclerosis obliterans compared to those of controls [54]. Thus, both of these studies suggest miR-210 as a biomarker for atherosclerosis. Eken et al. [41] reported that circulating miR-210 levels were lower in patients with unstable fiber caps compared to those with stable atherosclerotic plaques. This study not only indicates that miR-210 potentially plays a role in stabilizing atherosclerotic plaques but also suggests its feasibility as a marker for the risk of rupture of atherosclerotic plaques. Zhang et al. [55] identified six miRNAs, including miR-210, which were elevated in patients with ischemic heart disease. After comparing the Gensini score of each of the coronary heart disease (CHD) patients with the miRNA levels, they found that miR-210 expression was negatively correlated with the Gensini score, suggesting its potential utility as a marker for the severity of CHD.

### 3.2. Acute Coronary Syndrome

Acute coronary syndrome (ACS) is a more urgent and high-risk CVD than atherosclerosis, which can cause acute ischemia and severe damage through blockade of blood flow in cardiovascular tissue [56]. In an animal model of AMI, Hu et al. [43] injected a plasmid containing miR-210 into the experimental group. Postinjection, the left ventricular (LV) function of the experimental group was significantly healthier than that of the control group. They further reported an increase in angiogenesis and a reduction in the apoptotic rate and infarct size in the group injected with miR-210. Arif et al. [21] presented the same conclusion using mice of different germ lines. Fan et al. [39] proved the protective effect of miR-210 in AMI mice through histological evaluation and changes in cardiac function. Simultaneously, they reported upregulation of CD31, a typical protein involved in angiogenesis. Collectively, these findings confirmed that miR-210 has a significant protective effect on the cardiovascular system in AMI.

In a clinical study, Karakas et al. [57] followed up 1112 patients with coronary artery disease, including 430 patients with ACS and 682 patients with stable angina pectoris, who underwent coronary angiography for four years after surgery. They measured eight different miRNAs in the plasma, which were found to be associated with cardiovascular events, to evaluate the prognostic value of circulating miRNAs, showing that miR-210 expression is associated with cardiovascular-related death by approximately three standard deviations in ACS. This is the largest study conducted to date to evaluate the prognostic value of circulating miRNAs in coronary artery disease (CAD), providing significant and valuable clinical guidance. These studies reported the potential of miRNAs as biomarkers for risk estimation in CAD. Another study reported an increase of seven miRNAs, including miR-210, in the blood samples of AMI patients compared with healthy controls. In addition, the expression of HIF1-*α*/miR-210 as a group of hypoxia-marker genes in myocardial tissue was significantly associated with AMI-associated mortality [28].

### 3.3. Valvular Heart Disease

miR-210 is reportedly associated with valvular heart disease. Rosjo et al. [58] reported an increase in circulating miR-210 in patients with aortic stenosis compared with age- and sex-matched controls, and the NT-proBNP level showed the same increasing trend. The authors suggested that the increase of miR-210 may be derived from epithelial cells or the myocardium. During the subsequent 1287 d of follow-up, 26% (15) of the patients died, and a significant association was reported between higher circulating levels of miR-210 and increased mortality. This study suggests that miR-210 is a candidate index of risk and prognosis for aortic stenosis patients.

### 3.4. Pulmonary Arterial Hypertension

PAH is a potentially lethal and highly prevalent vascular disease characterized by increased pulmonary arterial pressure and lung vasculopathy [59]. A well-known cause of PAH is hypoxia and the activity of HIF-1*α* and HIF-2*α* [60]. Gou et al. [45] generated a PAH mouse model via chronic hypoxia, reported significant miR-210 upregulation, and showed that miR-210 upregulation plays an antiapoptotic role in hPASMCs. However, the responses of the pulmonary vascular tissue to hypoxia and HIF factors in PAH are partially associated with chronic repression of mitochondrial metabolism. ISCU1/ISCU2 plays an important role in PAH pathogenesis as a key component of mitochondrial metabolism, which could be regulated by miR-210 [17]. miR-210 is reportedly increased significantly in the small diseased pulmonary arterioles in human PAH lung tissues compared with arterioles observed in nondiseased human lung tissue. Circulating extracellular miR-210 is elevated in plasma samples adjacent to the pulmonary vascular space compared with samples from PAH-free individuals [17]. This study initially reports the progressive role of miR-210 to PAH through the inhibition of the ISCU1/2-FeS pathway through disease induction with SU5416. In turn, chronic miR-210 expression led to elevated right ventricular systolic pressure (RVSP) and induced pulmonary vascular remodeling [17]. Interestingly, SU5416, which can specifically induce PAH, induces the expression of miR-210 in whole lung tissue and in small pulmonary vessels, with no effects on miR-210 in the heart and other organs. In addition, levels of other reported miR-210 targets including E2F transcription factor 3 (E2F3) and Ephrin-A3 (*EFNA3*) remained unchanged, indicating an important role of ISCU1/2 in the specific activity of miR-210 in PAH. Therefore, the function of miR-210 in PAH is controversial.

### 3.5. Heart Failure

Because of the irreversible nature of myocardial injury, many CVDs eventually develop into heart failure. A plasma PCR array for patients with chronic heart failure revealed miR-210 upregulation [61]. Semenza reported that the mature miR-210 contents in mononuclear cells from blood samples of patients with congestive heart failure are associated with the existing diagnostic criteria for N-terminal probrain natriuretic peptide (NT-proBNP), which is recommended as a diagnostic tool for heart failure by the American Heart Association in 2005 and 2009 [26]. Moreover, although there was no significant correlation between miR-210 and NT-proBNP in NYHA-level 2 plasma, the miR-210 level was relatively lower in patients with improved NT-proBNP [62], indicating that miR-210 not only can be considered a marker for detecting the severity of heart failure but also could predict the prognosis of patients with heart failure.

### 3.6. Preeclampsia

Although there is no clear evidence of an association of miR-210 with primary or secondary hypertension, numerous studies have reported a significant increase in miR-210 in the blood of patients with preeclampsia (PE) [34, 63–66]. The disease manifests as high blood pressure during pregnancy, probably owing to hypoxia caused by insufficient placental perfusion. One method of generating a PE model is injecting the *TLR3* promoter in pregnant mice, which can increase blood levels of miR-210, which is regulated by both HIF1-*α*-dependent and HIF1-*α*-independent pathways [67]. Biro et al. [63] collected blood samples from patients with chronic hypertension, gestational hypertension, and mild or severe PE, along with a normal control group and a normal pregnancy group. Although they found a significant difference in the relative exosomal hsa-miR-210-3p expression levels between all disease subgroups compared to the normotensive group, only the PE subgroups showed a significant upregulation in miR-210. Using a PCR array to analyze the miRNA expression levels in PE patients, Murphy et al. [66] reported no apparent reduction in the levels of miR-210 and other related miRNAs after one year, suggesting that women with PE during pregnancy had persistent inflammation after childbirth. In addition to the blood, miR-210 upregulation was also detected in the placental tissue of PE patients. In addition, miR-210 reportedly regulates glucose metabolism and iron deposition in Swan 71 trophoblasts and BeWo choriocarcinoma cell lines through inhibition of ISCU [68]. A relationship between miR-210 expression and fetal weight was demonstrated in this clinical trial. Zhang et al. [34] reported that upregulated miR-210 expression in PE-related cells was involved in the regulation of *EFNA3* and/or *HOXA9* and other genes. In addition, the authors analyzed the expression of miR-210 in five different types of placentas and assessed mitochondrial function, showing that oxidative stress and reactive oxygen species (ROS) were produced in the placenta tissue cells of PE patients, thus increasing miR-210 levels and inhibiting mitochondrial-related ISCU, which potentially contributes to mitochondrial dysfunction [69]. These results were confirmed using the same cell line *in vitro*.

Hydroxysteroid 17*β*-dehydrogenase type 1 (HSD17B1), a steroid-producing enzyme that is primarily expressed in the placenta, is also one of the proteins regulated by miR-210. The mRNA and protein expression levels of HSD17B1 were significantly reduced in the placentas of PE patients with a concomitant reduction in plasma HSD17B1 protein levels. In addition, a prospective cohort study reported significantly reduced plasma HSD17B1 levels in pregnant women between 20–23 and 27–30 weeks before the onset of PE in comparison with subjects with a normal pregnancy. The authors concluded that expression of HSD17B1 in the placenta of PE patients is under the control of miR-210 and that plasma HSD17B1 levels are decreased before the onset of PE, suggesting HSD17B1 as a potential prognostic marker for PE [70].

### 3.7. Diabetes

Patients with diabetes often develop cardiac diseases [71, 72]. miR-210 is reportedly specifically upregulated in diabetes patients undergoing tests for heart failure. Although the levels of most of the detected miRNAs did not significantly differ between nondiabetes and diabetes patients with heart failure, the miR-210 level in the diabetes group was significantly higher [73]. Moreover, diabetes itself can cause changes in miR-210 expression in the body. Costantino et al. [74] reported several upregulated miRNAs, including miR-210, in a mouse model of streptozotocin-induced diabetes. miR-210 overexpression was further reported in Zucker diabetic fatty rats [75]. In clinical studies, elevated miR-210 has been detected in both the plasma and urine of adolescent type I diabetes mellitus patients, suggesting that high sugar itself can cause oxidative stress in cells [76]. Amr et al. [77] evaluated miR-210 expression in type 2 diabetes with or without CAD and reported that it is upregulated in both groups of diabetic patients. However, between these two groups, patients with CAD had a more significant miRNA change than those with diabetes alone.

## 4. The Protective Mechanism of miR-210 *In Vitro* CVD Models

As a prognostic miRNA in CVD, miR-210 regulates the number of genes and proteins related to cardiac models. Plasmids coding miR-210 injected into the myocardium of an AMI mouse model improved cardiac function, reduced infarct size, prevented endogenous myocardial apoptosis, and induced angiogenesis, suggesting that miR-210 potentially reduces cell death by regulating apoptosis in adult mice and promoting angiogenesis [21]. Further, Lu et al. [48] reported that HIF/miR-210/ISCU serves as an important signaling axis regulating mitochondrial metabolism, protecting rats from chronic hypoxia-induced PAH. These results indicate that miR-210 protects cells from stimulations through various mechanisms (Table 2).

### 4.1. Antiapoptosis

One of the primary protective effects of miR-210 is to inhibit apoptosis and promote cell survival. Li et al. [42] reported that miR-210 reduces apoptosis through inhibiting the caspase-8 pathway in H_2_O_2_-stimulated human umbilical vein endothelial cells (HUVECs). Similarly, Diao et al. [16] reported that miR-210 specifically inhibits the expression of the proapoptotic gene *BNIP3* (BCL2/adenovirus E1B 19 kDa protein-interacting protein 3), thereby preventing apoptosis in H9C2 cells. Li et al. [15] used oxidized low-density lipoprotein (ox-LDL) to induce miR-210 in human aortic endothelial cells (HAECs), demonstrating a negative correlation with PDK1 levels. Inhibition of miR-210 expression reportedly significantly reduced apoptosis in HAECs, whereas PDK1 overexpression reportedly reversed the proapoptotic effect of miR-210 through the P13K/AKT/mTOR pathway.

Several genes promote apoptosis through regulation of the cell cycle. APC is a part of the canonical Wnt signaling pathway that negatively regulates cell cycle progression by promoting *β*-catenin degradation, which could lead to cell death [21]. Highly expressed miR-210 in human carotid artery smooth muscle cells (HCtASMC) and human carotid artery endothelial cells (HCAEC) in a hypoxic condition can inhibit APC expression and consequently reduce apoptosis [41]. Another target of miR-210 is *E2F3*, a potential target for functional involvement in apoptosis, as its deletion leads to inhibition of apoptosis [13]. Under oxygen-glucose deprivation/reperfusion, upregulated miR-210 protects cardiomyocytes by inhibiting *E2F3* [46]. Moreover, Hu et al. [43] reported that hypoxia upregulates miR-210 in HL-1 cells and inhibits *EFNA3* and death-associated protein kinase 1 (*Dapk1*) to enhance the adaptation of HL-1 cells to hypoxia. These two genes reportedly play a negative role in the cell cycle and are related to apoptosis.

### 4.2. Proangiogenesis

When CVD affects the function of the cardiovascular system, an important mechanism of reducing injury is through angiogenesis. Angiogenesis is the process of formation of new blood vessels from preexisting vessels, which plays a role in cardiomyocyte growth, survival, and contractile function [78]. This involves several phenomena, including cell proliferation, differentiation, migration, and regulation of angiogenic factors. APC is involved in cell proliferation regulated by miR-210 according to Arif et al. [21]. Similar to APC, miR-210 also targets *E2F3*, which is closely related to cell proliferation [79]. Significant upregulation of miR-210 and downregulation of *E2F3* expression have been reported in a hypoxia model of human pulmonary artery smooth muscle cells (hPASMCs), and expression of *E2F3* was induced when miR-210 was inhibited [45]. The expression of MAP kinase phosphatase 1 (*MKP1*) in hPASMCs is also regulated by miR-210, and targeted inhibition of miR-210 reduces the proliferation of hPASMCs. Moreover, overexpression of miR-210 prevents hypoxia-induced *MKP1* expression with no effects on cell proliferation. Inhibition of *MKP1* by small interfering RNA abolished the miR-210-dependent prevention of cell proliferation under hypoxia [49]. Furthermore, in placental cytotrophoblast cells (CTs), Zhang et al. [34] detected the expression of cell proliferation-related genes *EFNA3* and homeobox A9 (*HOXA9*), which are regulated by miR-210. Hypoxia in CTs is a major cause of PE [80]. Interestingly, miR-210 downregulated the EFNA3 protein but not the mRNA in HUVECs [47]. Further, runt-related transcription factor 3 (RUNX3) was also identified as the direct target of miR-210 in HUVECs [51]. Overexpression of miR-210 downregulated RUNX3 and promoted the proliferation and migration of HUVECs, while overexpression of RUNX3 inhibited these effects. Sprouty-related enabled/vasodilator-stimulated phosphoprotein homology 1 domain-containing protein 2 (SPRED2) inhibits cell migration by negatively regulating the mitogen-activated protein kinase pathway. Moreover, human aortic smooth muscle cells (HASMCs) and HUVECs can be protected from ox-LDL by producing excess miR-210 [52]. This effect was achieved by inhibiting SPRED2. Furthermore, connective tissue growth factor (*Ctgf*), which is downregulated by miR-210, plays an important role in angiogenesis and fibrosis [43, 44]. Studies have reported the upregulation of Ctgf in the infarct zone of AMI rats in both cardiac fibroblasts and cardiac myocytes [81]. Therefore, we suggest that downregulated Ctgf by miR-210 could promote angiogenesis and reduce myocardium fibrosis.

*VEGF* is a topical gene that regulates cell proliferation and migration. It was reportedly upregulated in HUVECs in response to hypoxia [37, 47, 51]. Overexpression of miR-210 significantly upregulated VEGF and promoted the proliferation, migration, and invasiveness of HUVECs [21, 38]. Protein tyrosine phosphatase 1B (*Ptp1b*) is a 50 kDa enzyme on the endoplasmic reticulum, which negatively regulates the VEGF pathway in endothelial cells (ECs) [82]. As a target of miR-210, downregulated *Ptp1b* promotes angiogenesis and reduces apoptosis [43]. Furthermore, *Dapk1* is reportedly an miR-210 target that regulates cell migration. HGF, similar to VEGF, is also an angiogenesis-related protein, which has positive effects in HUVECs under hypoxia by regulating cell proliferation, migration, and differentiation [39].

In addition, Fasanaro et al. [50] identified regulator of differentiation 1 (ROD1) as a target of miR-210. ROD1 regulates cell differentiation, and its overexpression significantly inhibits cell proliferation. Although it does not have a distinct complementary sequence to that of miR-210, ROD1 is still regulated by miR-210. This indicates the existence of a class of proteins that are regulated by miRNAs without a seed sequence. This possibility has revealed novel avenues for future studies on miR-210.

### 4.3. Inhibition of Mitochondrial Metabolism

The shift in energy metabolism is another key to the survival of CMs under hypoxia or other stimulations. Iron is an essential metal necessary for normal cellular processes, including metabolism, heme synthesis, cell proliferation, and hypoxic response [17, 83]. The two major iron-containing molecules in the body are Fe/S clusters and heme, which are both involved in mitochondrial metabolism.

Iron-sulfur cluster scaffold protein (*ISCU*) is a key chaperone for the assembly of cellular ISCs and their transportation to enzymes, such as the mitochondrial respiratory complexes (complexes I, II, and III), which are responsible for mitochondrial respiration and energy production [14]. *ISCU* was first identified as a target gene of miR-210 in 2009 by Chan et al. in the human pulmonary arterial endothelial cells (HPAECs) [7]. Under hypoxic stress, miR-210 can target and downregulate ISCU in tumor cells, vascular endothelial cells, and many other cell types, thereby affecting the Krebs cycle, electron transport, ion metabolism, the production of ROS, and the function of mitochondria [84]. Sun et al. [14] reported that miR-210 regulates the shift in cellular energy metabolism during H_2_O_2_-induced oxidative stress in H9C2 CMs by targeting ISCU and indirectly regulating sirtuin 3 (SIRT3), which is also involved in energy metabolism. In hPASMCs, ISCU1/2 downregulation decreased Fe-S-dependent mitochondrial respiration and promoted a metabolic shift toward glycolysis for energy production. In a rat model of pulmonary hypertension, ISCU promoted the remodeling of pulmonary vessels by forming a positive feedback loop affecting the activity of mito-KATP and the proliferation of hPASMCs [48].

Further, miR-210 decrease heme levels by targeting ferrochelatase (FECH) in CMs, which is induced by HIF [85]. The final and rate-limiting step occurs in the mitochondria, where FECH inserts an iron molecule into protoporphyrin IX to generate heme. Qiao et al. [83] reported that overexpression of ISCU does not affect changes mediated by miR-210 in heme and FECH, showing that regulation of FECH by miR-210 is independent of ISCU. However, FECH levels increased without reversal via miR-210 knockdown during hypoxia, suggesting that the effects of miR-210 on heme are restricted to normoxic conditions and that the pathway is overridden during hypoxia. Therefore, further studies are required to determine the association between miR-210 and heme.

### 4.4. Inhibition of Inflammation

During hypoxia and other stimulations, immune responses eliminate damaged cells and tissues. However, excessive immune response can lead to inflammatory reactions and severe damage to heart tissue. Since miR-210 could be activated by NF-*κ*B, miR-210 regulates inflammation by targeting several key genes in inflammatory pathways.

STAT6, an important member of the STAT family, is a key responder to the stimulation by interleukin-4 (IL-4) and IL-13 in the differentiation of T helper 2 cells [86]. Kopriva et al. [67] reported that TLR3-induced placental miR-210 downregulated the STAT6/interleukin-4 pathway, thus protecting PE mice from inflammatory injury.

In adult mice with acute ischemic brain injury, miR-210 inhibition significantly downregulated proinflammatory cytokines (TNF-*α*, IL-1*β*, and IL-6) and chemokines (CCL2 and CCL3) but had no significant effect on anti-inflammatory factors (TGF-*β* and IL-10) [87]. Moreover, antiphospholipid antibody- (aPL-) induced inflammation of trophoblast cells resulted in significant upregulation of miR-210 [88]. The TLR4 antagonist downregulated miR-210 in this model, thus showing the TLR4-dependent regulation of miR-210. These results revealed a close association between miR-210 and inflammation, providing potential issues related to CVD.

## 5. Therapeutic Potential of miR-210

Spinetti et al. [89] reported a trend of increasing miR-210 levels in the plasma of long-living individuals (LLIs; aged >90 years) compared to those of younger sex-matched controls. Interestingly, they reported miR-210 upregulation in the nonhealthy group of LLIs compared to healthy LLIs. This difference may be associated with the migration of miR-210-stimulated cells and may also be associated with the regulation of miR-210 during mitochondrial metabolism and ROS production. In this process, the two predicted target genes of miR-210, *CXCR4* and *BPIFB4*, displayed a significant correlation with miR-210 expression. Thus, these two genes may provide a unique expression signature of health conservation with aging, independent of lifespan. Indeed, CXCR4 is considered a marker of healthy aging. Further studies involving pregnant women chronically living at high altitudes in comparison with those living at a sea level displayed an increase in the level of miR-210 expression [40], thus providing evidence regarding miR-210 as a hypoxia-protective miRNA in healthy individuals.

Recent animal experiments have identified certain drugs that can upregulate miR-210. Saffron has long been used in traditional medicine owing to its various pharmacological effects such as antioxidant, anticancer, anti-inflammatory, and antiatherosclerotic effects [90]. Crocin is an active ingredient in saffron, considered responsible for the beneficial effects. Ghorbanzadeh et al. [91] administered a single dose of crocin (50 mg/kg) and/or voluntary exercise for eight weeks in a rat model and reported an increase in miR-210, AKT, and ERK1/2 levels in the myocardial tissues compared to the control. Moreover, the crocin+exercise group displayed significant changes in various indicators. Together, these results suggest that saffron and exercise can be combined as a new potential therapeutic or preventive strategy to overcome certain CVDs.

Another study reported that pretreatment of a mouse model of AMI with Huoxue Anxin Recipe (HAR) upregulated miR-210 expression, while the untreated group displayed a relative downregulation of miR-210 [38]. Moreover, the ejection fraction, fractional shortening of the mice, in the HAR treatment group was higher than that in the AMI-untreated group, and the infarct size was smaller than that in the untreated group. Furthermore, the upregulation of vascular endothelial growth factor suggested that these effects might be achieved by promoting myocardium angiogenesis. Olmesartan is a member of angiotensin II receptor blockers, which plays an important role in treating hypertension [92]. When evaluating the protective function of olmesartan in a rat model of spontaneously hypertensive myocardial ischemia reperfusion, Lu et al. [93] reported that miR-210 is a relatively independent indicator of myocardial protection.

Owing to the unique nature of the heart tissue, the damage caused by CVD is usually irreversible. Although current therapeutic interventions for CHD can improve clinical outcomes and prolong lifespans, they are essentially palliative in nature because they fail to address the underlying issues of the loss of CMs. Recent studies have attempted to regenerate heart tissue by inducing the differentiation of various stem cells. Early stem cell transplantation is directly related to the recovery of late cardiac function [94]. However, the low survival rate of stem cells after transplantation has been a major obstacle to their clinical application [95]. Intermittent hypoxia can protect cells via the HIF1-*α* pathway [96], and miR-210 can affect cell migration, differentiation, and angiogenesis and inhibit apoptosis. Accordingly, Kim et al. [95] performed two rounds of 30 min hypoxic preconditioning on bone marrow-derived mesenchymal stem cells (MSCs), which were then injected into the myocardium of an AMI model. miR-210 was significantly upregulated in the group with preconditioned MSCs, and the cell viability was improved after implantation in the rats. These effects may have been achieved by inhibiting the apoptotic protein caspase-8. In another experiment, miR-210 expression was induced in MSCs, which promoted the survival of CMs in coculture, and miR-210 was induced and transferred by exosomes in both cell types [97]. In the same cell line, Wen et al. [98] stimulated miR-210 via sevoflurane preconditioning, thus exerting the same effect as that of transplanted stem cells. Ma et al. [99] reported that endothelial progenitor cells can protect endothelial cells through exosome-derived miR-210, which promotes this protective effect by improving mitochondrial function. In a study on human endothelial stem cells (hESCs), Kane et al. [100] reported elevated miR-210 during differentiation of hESCs into functional vascular endothelial cells, suggesting that miRNA-210 can promote angiogenesis. Xu et al. [101] conducted a detailed study on the protective mechanism of miR-210 overexpression in MSCs, showing that it improved the survival of MSCs under oxidative stress through antioxidation and the activation of the c-Met pathway. These results provide a rationale for stem cell-based therapy. The same prosurvival effect was also reported in myocardial progenitor cells (MPCs), displaying less CM apoptosis, enhanced angiogenesis, and improved LV ejection in the miR-210-injected group in comparison with hearts injected with cell culture medium [102]. A plasmid harboring HIF1-*α* and CPCs were simultaneously implanted into the injured myocardium of rats. The infarct size was relatively small, and the ejection fraction of the rats was higher after AMI compared to the control group. These findings indicate that the implanted HIF1-*α* can prolong the survival of transplanted CPCs, preventing cardiac remodeling, reducing the infarct size, and promoting angiogenesis. Furthermore, this function is at least partially mediated by miR-210 [103]. Finally, Emanueli et al. [104] reported elevated plasma miR-210 levels in patients undergoing coronary artery bypass grafting, which was primarily focused on the exosomes, confirming that miR-210 is transferred via exosomes in the repair of myocardial injury.

## 6. Conclusion

Emerging evidence suggests that miR-210 can be considered an independent indicator of the extent of cellular damage and for severity assessment of various clinical CVDs. In addition, miR-210 can also predict the prognosis of diseases such as atherosclerosis and PE and can guide the development of follow-up treatment strategies. miR-210 is also expected to play a protective role in heart tissue, especially during stem cell transplantation and heart transplantation. Although negative functions have been reported in some situations associated with CVDs, miR-210 upregulation is clearly associated with protection of cardiovascular cells.

In conclusion, miRNA-210, as a recognized hypoxic miRNA, offers great potential in treating CVDs. Thus, further studies are required to investigate the cardiovascular protective mechanism of miR-210 and its future application.

## Figures and Tables

**Table 1:** Genes regulated by miR-210 in the CVD model.

Target	Function	Cell line	References
APC	Antiproliferation, proapoptosis, and cell cycle inhibition	CM, HCtASMC, and HCAEC	[21, 41]
BNIP3	Proapoptosis	H9C2	[16]
CASP8AP2	Proapoptosis	HUVEC	[42]
Ctgf	Antiangiogenesis, profibrosis	HL-1, cardiac fibroblast	[43, 44]
Dapk1	Cell cycle inhibition, proapoptosis, and antimigration	HL-1	[43]
E2F3	Cell cycle inhibition, proapoptosis	hPASMC, CM	[45, 46]
EFNA3	Antiangiogenesis, proapoptosis	CT, HL-1, and HUVEC	[34, 43, 47]
HGF	Proangiogenesis, proproliferation, promigration, and prodifferentiation	HUVEC	[39]
HOXA9	Antiproliferation	CT	[34]
ISCU	Mitochondrial metabolism progression	H9C2, hPASMC, and hPAEC	[7, 14, 17, 48]
MKP1	Antiproliferation	hPASMC	[49]
PDK1	Proapoptosis	HAEC	[15]
Ptp1b	Antiangiogenesis, proapoptosis	HL-1	[43]
ROD1	Antidifferentiation (seedless)	HUVEC	[50]
RUNX3	Antiproliferation, antimigration	HUVEC	[51]
SPRED2	Antimigration	HASMC, HUVEC	[52]
VEGF	Proangiogenesis, promigration, and proinvasion	HUVEC	[21, 37, 38, 47, 51]

**Table 2:** Protection mechanism of microRNA-210 in hypoxic cells.

Function	Gene	Reference
Antiapoptosis	APC/BNIP3/CASP8AP2/Dapk1/E2F3/EFNA3/PDK1/Ptp1b	[15, 16, 21, 34, 41–43, 45, 46]
Proangiogenesis	Ctgf/HGF/Ptp1b/VEGF	[37–39, 43, 47, 51]
Proproliferation	APC/HGF/ HOXA9/MKP1/RUNX3	[21, 34, 39, 41, 49, 51]
Promigration	Dapk1/HGF/KCMF1/RUNX3/SPRED2/VEGF	[21, 37–39, 43, 47, 51–53]
Prodifferentiation	HGF/ROD1	[39, 50]
Cell cycle progression	APC/Dapk1/E2F3/EFNA3	[21, 34, 39, 41, 43, 45–47]
Antifibrosis	Ctgf	[43, 44]
Mitochondrial metabolism inhibition	ISCU	[7, 14, 48]
Others	HSD17B1	[70]
